# Contrasting Dihydronaphthoquinone Patterns in Closely Related *Drosera* (Sundew) Species Enable Taxonomic Distinction and Identification

**DOI:** 10.3390/plants10081601

**Published:** 2021-08-04

**Authors:** Jan Schlauer, Siegfried R. H. Hartmeyer, Irmgard Hartmeyer, Tuulikki Seppänen-Laakso, Heiko Rischer

**Affiliations:** 1 The Center for Plant Molecular Biology (ZMBP), University of Tuebingen, Auf der Morgenstelle 32, D-72076 Tuebingen, Germany; 2 Independent Researcher, Wittlinger Str. 5, D-79576 Weil am Rhein, Germany; s.hartmeyer@t-online.de (S.R.H.H.); irmgard@hartmeyer.de (I.H.); 3 VTT Technical Research Centre of Finland Ltd., Tietotie 2, FIN-02044 Espoo, Finland; tuulikki.seppanen-laakso@vtt.fi

**Keywords:** *Drosera*, droseraceae, naphthoquinones, chemotaxonomy

## Abstract

Dihydronaphthoquinones are described as constituents of sundews (*Drosera*), Venus flytraps (*Dionaea*), and dewy pines (*Drosophyllum*) for the first time. As in the corresponding naphthoquinones, these reduced derivatives may occur in two regio-isomeric series distinguished by the relative position of a methyl group (at position 2 or 7 in the naphthalene skeleton), depending on the taxon. Species producing plumbagin (2-methyljuglone, **1**) do commonly contain the corresponding dihydroplumbagin (**5**), while species containing ramentaceone (7-methyljuglone, **2**) also contain dihydroramentaceone (7-methyl-β-dihydrojuglone, **6**). So far, only few species containing plumbagin (**1**) and dihydroplumbagin (**5**) additionally form dihydroramentaceone (**6**) but not ramentaceone (**2**). Thus, subtle but constant differences in the chemism of closely related and morphologically similar species reliably define and distinguish taxa within *D*. sect. *Arachnopus*, which is taken to exemplify their chemotaxonomic utility. The joint presence of quinones and hydroquinones allows observations and predictions on the chemical structures and the reactions of these intriguing natural products.

## 1. Introduction

**Scheme 1:**
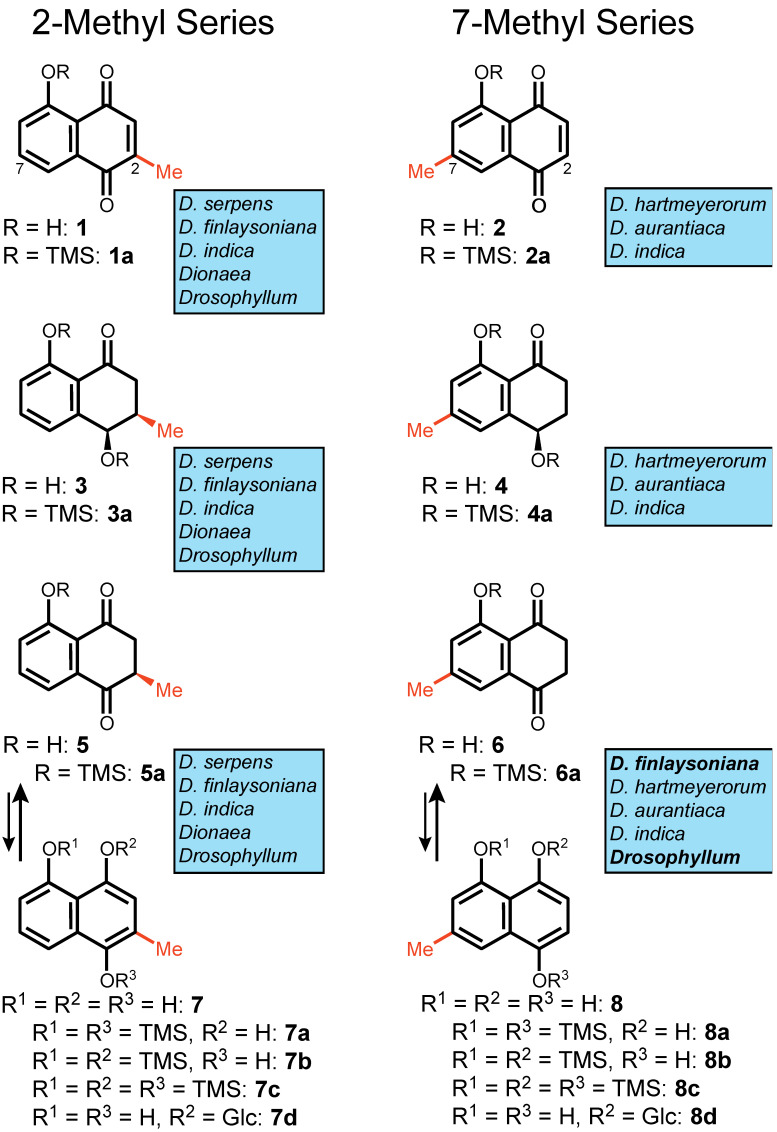
Naphthoquinone derivatives in *Drosera* and related genera. ***Bold italic*** script indicates taxa where dihydroquinones contrast with their “proper” series.

Following contemporary practice, several segregate taxa are recognized [1] in *Drosera* sect. *Arachnopus* Planch., a group of sundews (*Drosera* L., Droseraceae) that previously only contained a single, collective species *D. indica* L. s. lat. [2] Previous research mainly based on TLC screening [3,4,5,6,7,8] has demonstrated a rather unexpected diversity in the naphthoquinones that are characteristic for the different species now distinguished in this group. While a few Australian species (e.g., *D. hartmeyerorum* J. Schlauer and *D. aurantiaca* A. Lowrie) contain ramentaceone (7-methyljuglone, **2**) together with the corresponding tetralone shinanolone (**4**) [5,6], most species (including *D. finlaysoniana* Wall. ex Arn. and *D. serpens* Planch.—accessions of both species from Australia, Indochina, and Eastern Asia have been investigated) contain the regio-isomer plumbagin (2-methyljuglone, **1**) together with isoshinanolone (**3**) [5,6,8]. *D. indica* s. str. (accessions from Asia and Africa have been investigated) contains all four compounds (**1**–**4**, Scheme 1) in the same plant [6]. It has thus been demonstrated by multiple samples that naphthoquinone patterns are stable and characteristic of the individual species across their entire geographic range.

Upon closer inspection of the respective TLC chromatograms, all of the *D. finlaysoniana* samples stood out because of the consistent presence of an additional constituent that distinguishes this species even from its relatives (e.g., *D. serpens*) that share the same major naphthoquinone (Figure S11). In this paper, we identify this additional metabolite and evaluate its chemotaxonomic potential both within *Drosera* and among related genera and families.

GC-MS was used for the structural elucidation of the additional constituents coming directly from crude petroleum ether extracts from the fresh leaves of several representatives of *Drosera* sect. *Arachnopus* and of *Dionaea muscipula* Soland. ex J. Ellis (Droseraceae, Venus’s flytrap) and *Drosophyllum lusitanicum* (L.) Link (Drosophyllaceae, dewy pine), which were chosen for outgroup comparison.

## 2. Results

**Figure 1:**
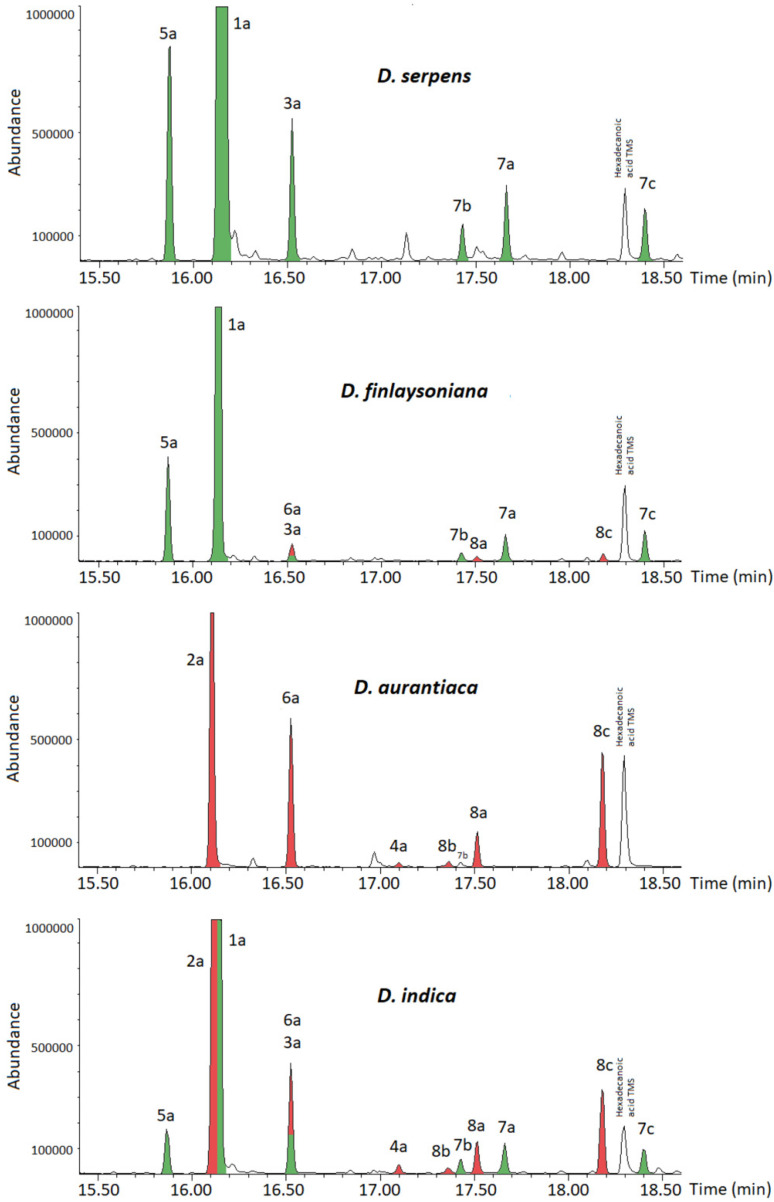
GC-MS TIC chromatograms of TMS-derivatives of metabolites in petroleum ether extracts of four sundew species. Metabolites of the 2-methyl series are marked in green, and those of the 7-methyl series are marked red.

Results are summarized in Table 1. As expected from previous TLC results, naphthoquinones were detected in all samples: plumbagin (**1**) in *D. finlaysoniana*, *D. serpens*, *Dionaea* and *Drosophyllum*; ramentaceone (**2**) in *D. hartmeyerorum* and *D. aurantiaca*; and both isomers in *D. indica*. In addition to the naphthoquinones, the tetralone isoshinanolone (**3**) was detected in *D. serpens*, *D. finlaysoniana*, *D. indica*, *Dionaea,* and *Drosophyllum*, while shinanolone (**4**) was the tetralone associated with ramentaceone (**2**) in *D. hartmeyerorum*, *D. aurantiaca*, and *D. indica*. In addition, a metabolite (*R*_t_ = 16.55 min, *m/z* 190) was detected in *D. finlaysoniana*, *D. hartmeyerorum*, *D. aurantiaca*, *D. indica*, and *Drosophyllum* but not in *D. serpens* and *Dionaea*. More detailed study disclosed the presence of another metabolite (*R*_t_ = 15.90 min, *m/z* 190) in *D. finlaysoniana*, *D. indica* and *D. serpens* that was not entirely chromatographically resolved from plumbagin (**1**). It is only after TMS-derivatization that all of the metabolites were separated clearly (Figure 1), and the respective mass spectra (Table S1 in Supplementary Materials) identify the additional metabolites as dihydronaphthoquinones dihydroplumbagin (**5**, *R*_t_ = 15.90 min) and dihydroramentaceone (**6**, *R*_t_ = 16.55 min), respectively. The extract of *D. serpens* only contained plumbagin (**1**) and dihydroplumbagin (**3**) but no dihydroramentaceone (**6**).

In addition to these naphthoquinone derivatives, different compounds (characterized by R_t_ = 14.66 min, *m/z* 204; R_t_ = 16.57 min, *m/z* 232; R_t_ = 16.64 min, *m/z* 216; R_t_ = 18.08 min, *m/z* 246, respectively) have been detected in the extracts from *Drosophyllum* but have not further identified. Possibly some of these represent derivatives of naphthoic acids that have been isolated from *Drosophyllum* and the related genus *Ancistrocladus* Wall. (Ancistrocladaceae) before [9,10].

Neither dihydronaphthoquinone has been isolated from *Drosera* or *Drosophyllum* before, but dihydroplumbagin (**5**) had been previously obtained and characterized from *Juglans* L. (Juglandaceae) species [11], and dihydroplumbagin (**5**) and dihydroramentaceone (**6**) were already known from *Diospyros maritima* Blume (Ebenaceae) [12].

## 3. Discussion

The mass spectra of the TMS derivatives of both dihydronaphthoquinones indicate that the diketo-tautomers (assigned to structures **5a** and **6a**, characterized by *m/z* 247) are predominant in the petroleum ether extract. In contrast, the naphthotriol-tautomers (**7** and **8**) were not detected in underivatized samples but may be represented by their TMS derivatives (**7c** and **8c**, characterized by *m/z* 406; **7a** and **8a**; *m/z* 318; **7b** and **8b**, *m/z* 319) that are less abundant (following the thermodynamic ratio of about 1:4) [13]. This corresponds well with the fact that natural dihydroplumbagin (**5**) isolated with chloroform from *Diospyros maritima* retains the same configuration (2*R*) as the corresponding position (3*R*) in isoshinanolone (**3**) from the same plant [12], so tautomerism and subsequent racemization may be limited in aprotic solvents. The hypothetical naphthotriol tautomers (**7** and **8**) are possibly able to form quinhydrones with the corresponding naphthoquinones. In the case of the 2-methyl derivative (**7**), this would have to occur in a fashion that conserves stereochemistry at C-2 upon the release of dihydroplumbagin (**5**) from the quinhydrone, even if the latter underwent an intermolecular proton transfer. Apparently, no mixed quinhydrones are formed from plumbagin (**1**) and dihydroramentaceone (**6**), or at least no intermolecular proton transfer occurs in such complexes. Otherwise, ramentaceone (**2**) would have been detected in *D. finlaysoniana*. The di-TMS derivatives with a phenolic hydroxyl group in *peri*-position to an *O*-TMS (**7a** and **8a**) lose a methyl group from TMS together with a proton (assumedly from the phenol) in MS, while those with a more remote hydroxyl group (**7b** and **8b**) only lose a methyl group. This may indicate that the resulting fragments from the former (**7a** and **8a**) can form dioxydimethylsilane bridges between C-4 and C-5.

While dihydroplumbagin (**5**) can readily be construed as a metabolic precursor of plumbagin [6], the simultaneous presence of dihydroramentaceone (**6**) in *D. finlaysoniana*, that is otherwise devoid of any ramentaceone metabolites is outright surprising, especially if compared to *D. serpens,* which shares the plumbagin derivatives (**1** and **3**) but obviously lacks any trace of dihydroramentaceone (**6**) in all geographically diverse specimens that have been investigated to date.

A minor regio-isomer of a dihydronaphthoquinone derivative was observed before in *D. intermedia* Hayne that contains plumbagin (**1)** as the main constituent along with plumbaside-C (**7d**) and trace amounts of rossoliside (**8d**), while ramentaceone (**2**) was only detected after hydrolysis and oxidation [14]. The recurrent pattern of plumbagin (**1**) as the main constituent and minor amounts of reduced derivatives from only the 7-methyl series, e.g., dihydroramentaceone (**6**) or its 4-*O*-glucoside (**8d**), may represent a plesiomorphic character state in the genus *Drosera* because it is found in the related genus *Drosophyllum* and in species that are supposed to occupy the basal positions in their respective sections: *D. finlaysoniana* in *D*. sect. *Arachnopus*, and *D*. *intermedia* in *D*. sect. *Drosera*.

The joint presence of plumbagin (**1**) and ramentaceone (**2**) has repeatedly been found in species of known or assumed hybrid origin [4,15,16]. In species that display a similar “hybrid” quinone pattern but that lack quinone-heterogenous candidate parent species, e.g., *D. regia* Stephens (*D*. sect. *Regiae*) [2,16], *D. stenopetala* Hook. f. (*D*. sect. *Psychophila*) [16], *D. ultramafica* A. Fleischm et al. (*D*. sect. *Drosera*) [7], or *D. indica* (*D*. sect. *Arachnopus*) [2,6,7], this condition may alternatively be derived from a predisposition similar to *D. finlaysoniana* or *D. intermedia* if the biosynthesis of the minor isomer is enhanced and its oxidation to the respective quinone is enabled or facilitated.

In contrast, *D. serpens* and its close relatives in which exclusively 2-methyl derivatives have been detected probably have a different genetic background, leading to higher regioselectivity.

The opposite extreme is observed in *D. hartmeyerorum* and *D. aurantiaca,* which exclusively contain derivatives of the 7-methyl series.

## 4. Materials and Methods

### 4.1. General Experimental Procedures

For GC-MS analyses of underivatized samples, a Gerstel MPS 2XL autosampler was used to inject (split ratio 20:1) the aliquots into an Agilent 7890B GC-system combined with an Agilent 5977B GC/MSD detector. The MS data at a mass range of 25–550 amu were collected and processed with Agilent MassHunter software. GC-MS runs were performed on an HP-5MS ultra inert silica capillary column (30 m, 0.250 mm ID, phase thickness 0.25 µm) using an oven temperature program from 50 °C to 260 °C at a rate of 10 °C min^−1^. The injector, MS source, and MS quadrupole temperatures were 260, 230, and 130 °C, respectively.

For the GC-MS analyses of TMS derivatives, a Gerstel Maestro MPS 2 sampling system (Gerstel GmbH&Co. KG, Mühlheim am der Ruhr, Germany) was used to inject the aliquots into an Agilent 7890A GC combined with a 5975C mass selective detector, equipped with a DB-5 silica capillary column (30 m, 0.250 mm, 0.25 µm, Restek, Bellefonte, PA, USA), and the oven temperature program was from 80 °C (1 min) to 100 °C (5 °C min^−1^) and to 250 °C (10 °C min^−1^; 10 min). The split ratio was 10:1, and the data were collected at a mass range of 35–800 amu. The injector temperature was 250 °C, and those of the MS source and the MS quadrupole were 230 and 150 °C, respectively.

Thin-layer chromatography analysis was conducted on silica gel 60 F_254_ plates (Macherey-Nagel, Düren, Germany) with toluene as the mobile phase. The spots were visualized using UV fluorescence (tetralones and dihydronaphthoquinones) and after exposure to NH_3_ fumes from a saturated aq. soln. (naphthoquinones).

### 4.2. Plant Material

Fresh leaves from plants in cultivation (the annual representatives of *D.* sect. *Arachnopus* were grown from seed, *Dionaea* and *Drosophyllum* were obtained from horticultural trade) were used for extraction and analysis. Authentic specimens prepared at the sampling date (October 2020) were deposited in the first author’s herbarium. Taxonomic identification of all samples was performed and confirmed by the authors. Their respective accession numbers are listed in Table 1.

### 4.3. Extraction and Isolation

Fresh leaf material (24–117 mg) was extracted with 500 µL petroleum ether (boiling range 60–80 °C, Emsure^®^, Merck KGaA, Darmstadt, Germany) for 1.5 h at 20 °C. The crude extracts were analyzed directly by TLC for the screening of volatile constituents (a representative chromatogram is available as Supplementary Material) and by GC-MS for structure determination.

The extracts were concentrated, and 2 µL aliquots were analyzed using GC-MS. After direct runs, the samples were evaporated under nitrogen flow and were dissolved into dichloromethane and trimethylsilylated with MSTFA (*N*-Methyl-*N*-trimethylsilyltrifluoro-acetamide, Pierce, Rockford, IL, USA) containing 0.1% trimethylchlorosilane (TMCS). The samples (2 µL aliquots) were analyzed through GC-MS (original spectra are available as Supplementary Material).

Identification of the compounds was based on retention times, library comparison (NIST ’08, Scientific Instrument Services, Inc., Ringoes, NJ, USA), and literature data [12].

*Plumbagin* (**1**): C_11_H_8_O_3_, EIMS *m/z* 188 [M]^+^ (100), 173 [M-CH_3_]^+^ (30), 160 [M-CO]^+^ (25), 132 (30), 131 [M-C_3_H_5_O]^+^ (50), 121 [M-C_4_H_3_O]^+^ (15), 120 [M-C_4_H_4_O]^+^ (25), 103 [M-C_4_H_5_O_2_]^+^ (10), 92 [M-C_5_H_4_O_2_]^+^ (40), 77 (15), 63 (40).

*Ramentaceone* (**2**): C_11_H_8_O_3_, EIMS *m/z* 188 [M]^+^ (100), 187 [M − H]^+^ (30), 173 [M − CH_3_]^+^ (10), 160 [M − CO]^+^ (15), 134 [M − C_3_H_2_O]^+^ (20), 132 (30), 131 [M − C_3_H_5_O]^+^ (30), 106 [M − C_4_H_2_O_2_]^+^ (20), 104 (10), 103 [M − C_4_H_5_O_2_]^+^ (10), 78 (15), 77 (25), 63 (15), 62 (10), 51 (25).

*Isoshinanolone* (**3**): C_11_H_12_O_3_, EIMS *m/z* 192 [M]^+^ (70), 177 [M − CH_3_]^+^ (20), 174 [M − H_2_O]^+^ (10), 150 [M − C_3_H_6_]^+^ (40), 149 [M − C_2_H_3_O]^+^ (25), 131 [M − C_2_H_5_O_2_]^+^ (20), 122 [M − C_3_H_2_O_2_]^+^ (45), 121 [M − C_4_H_7_O]^+^ (100), 115 (10), 93 (25), 77 (20), 65 (30), 51 (20).

*Shinanolone* (**4**): C_11_H_12_O_3_, EIMS *m/z* 192 [M]^+^ (70), 177 [M − CH_3_]^+^ (10), 174 [M − H_2_O]^+^ (10), 164 [M − C_2_H_4_]^+^ (20), 149 [M − C_2_H_3_O]^+^ (15), 135 [M − C_3_H_5_O]^+^ (100), 107 [M − C_4_H_5_O_2_]^+^ (20).

*Dihydroplumbagin* (**5**): C_11_H_10_O_3_, EIMS *m/z* 190 [M]^+^ (85), 175 [M − CH_3_]^+^ (100), 162 [M − CO]^+^ (20), 147 [M − C_2_H_3_O]^+^ (20), 120 [M − C_4_H_6_O]^+^ (60), 92 [M − C_5_H_6_O_2_]^+^ (40), 63 (15) (Figure S1).

*Dihydroramentaceone* (**6**): C_11_H_10_O_3_, EIMS *m/z* 190 [M]^+^ (100), 175 [M − CH_3_]^+^ (10), 162 [M − CO]^+^ (15), 134 [M − C_3_H_4_O]^+^ (55), 106 [M − C_4_H_4_O_2_]^+^ (20) (Figure S2).

*5-O-Trimethylsilyl-plumbagin* (**1a**): C_14_H_16_O_3_Si, EIMS *m/z* 245 [M − CH_3_]^+^ (100), 217 [M − CH_3_ − CO]^+^ (30), 186 (10), 115 (10) (Figure S3).

*5-O-Trimethylsilyl-ramentaceone* (**2a**): C_14_H_16_O_3_Si, EIMS *m/z* 245 [M − CH_3_]^+^ (100), 217 [M − CH_3_ − CO]^+^ (20), 187 (10), 115 (10) (Figure S4).

*4,8-Di-(O-trimethylsilyl)-isoshinanolone* (**3a**): C_17_H_28_O_3_Si_2_, EIMS *m/z* 321 [M − CH_3_]^+^ (5), 231 [M − CH_3_ − C_3_H_10_OSi]^+^ (100), 216 [M − 2CH_3_ − C_3_H_10_OSi]^+^ (20), 201 [M − 3CH_3_ − C_3_H_10_OSi]^+^ (10), 186 [M − 4CH_3_ − C_3_H_10_OSi]^+^ (5) (Figure S5).

*4,8-Di-(O-trimethylsilyl)-shinanolone* (**4a**): C_17_H_28_O_3_Si_2_, EIMS *m/z* 321 [M − CH_3_]^+^ (5), 231 [M − CH_3_ − C_3_H_10_OSi]^+^ (100), 216 [M − 2CH_3_ − C_3_H_10_OSi]^+^ (20), 201 [M − 3CH_3_ − C_3_H_10_OSi]^+^ (10), 186 [M − 4CH_3_ − C_3_H_10_OSi]^+^ (5) (Figure S6).

*5-O-Trimethylsilyl-dihydroplumbagin* (**5a**): C_14_H_18_O_3_Si, EIMS *m/z* 247 [M − CH_3_]^+^ (100), 219 [M − CH_3_ − CO]^+^ (10) (Figure S7).

*5-O-Trimethylsilyl-dihydroramentaceone* (**6a**): C_14_H_18_O_3_Si, EIMS *m/z* 247 [M − CH_3_]^+^ (100), 219 [M − CH_3_ − CO]^+^ (10) (Figure S8).

*1,5-Di-(O-trimethylsilyl)-2-methyl-naphtho-1,4,5-triol* (**7a**): C_17_H_26_O_3_Si_2_, EIMS *m/z* 318 [M − CH_4_]^+^ (100), 288 [M − CH_4_ − 2CH_3_]^+^ (20), 273 [M − CH_4_ − 3CH_3_]^+^ (10) (Figure S9).

*4,5-Di-(O-trimethylsilyl)-2-methyl-naphtho-1,4,5-triol* (**7b**): C_17_H_26_O_3_Si_2_, EIMS *m/z* 319 [M − CH_3_]^+^ (100), 245 (15), 217 (10).

*2-Methyl-1,4,5-tri-(O-trimethylsilyl)-naphtho-1,4,5-triol* (**7c**): C_20_H_34_O_3_Si_3_, EIMS *m/z* 406 [M]^+^ (100).

*1,5-Di-(O-trimethylsilyl)-7-methyl-naphtho-1,4,5-triol* (**8a**): C_17_H_26_O_3_Si_2_, EIMS *m/z* 318 [M − CH_4_]^+^ (100), 288 [M − CH_4_ − 2CH_3_]^+^ (20), 273 [M − CH_4_ − 3CH_3_]^+^ (10) (Figure S10).

*4,5-Di-(O-trimethylsilyl)-7-methyl-naphtho-1,4,5-triol* (**8b**): C_17_H_26_O_3_Si_2_, EIMS *m/z* 319 [M − CH_3_]^+^ (100), 245 (15), 217 (10).

*7-Methyl-1,4,5-tri-(O-trimethylsilyl)-naphtho-1,4,5-triol* (**8c**): C_20_H_34_O_3_Si_3_, EIMS *m/z* 406 [M]^+^ (100).

## Figures and Tables

**Table 1:** Naphthoquinone derivatives identified in petroleum ether extracts of the plant material investigated in this study.

Species (Provenance, Accession No.)		2-Methyl Series			7-Methyl Series		
		Plumbagin (1)	Dihydroplumbagin (5)/(7)	Isoshinanolone (3)	Ramentaceone (2)	Dihydroramentaceone (6)/(8)	Shinanolone (4)
*D. serpens* (Australia, 2020_101)		+	+	+	-	-	-
*D. finlaysoniana* (Tropical Asia, 2020_102)		+	+	+	-	+ *	-
*D. hartmeyerorum * (Australia, 2020_103)		-	-	-	+	+	+
*D. aurantiaca * (Australia, 2020_104)		-	-	-	+	+	+
*D. indica * (Tropical Asia, 2020_105)		+	+	+	+	+	+
*Dionaea muscipula* (USA, 2020_106)		+	+	+	-	-	-
*Drosophyllum lusitanicum* (SW Europe, 2020_107)		+	+	+	-	+ *	-
*R*_t_ (GC) [min]		14.49	14.47	15.46	14.70	15.56	15.97?
M^+^ [*m/z*]		188.0	190.1	192.1	188.0	190.1	192.1
*R*_t_ (GC) [min]; characteristic MS signal [*m*/*z*] of TMS-Derivatives	TMS	16.15; 245.1 (**1a**)	15.88; 247.1 (**5a**)	-	16.13; 245.1 (**2a**)	16.54; 247.1 (**6a**)	
	TMS_2_	-	17.65; 318.2 (**7a**) 17.43; 319.1 (**7b**)	16.52; 231.1 (**3a**)	-	17.52; 318.2 (**8a**) 17.37; 319.1 (**8b**)	17.10; 231.1 (**4a**)
	TMS_3_	-	18.41; 406.3 (**7c**)	-	-	18.18; 406.3 (**8c**)	-

Numbers in bold refer to compounds in Scheme 1 and in the text. * Detection of dihydroramentaceone in species that otherwise lack representatives of the 7-methyl series.

## Data Availability

All data, tables and figures in this manuscript are original except in cases indicated by respective references.

## Supplementary Materials

The following are available online at https://www.mdpi.com/article/10.3390/plants10081601/s1, Figure S1: MS of dihydroplumbagin (**5**), Figure S2: MS of dihydroramentaceone (**6**), Figure S3: MS of TMS derivative compound **1a**, Figure S4: MS of TMS derivative compound **2a**, Figure S5: MS of TMS derivative compound **3a**, Figure S6: MS of TMS derivative compound **4a**, Figure S7: MS of TMS derivative compound **5a**, Figure S8: MS of TMS derivative compound **6a**, Figure S9: MS of TMS derivative compound **7a**, Figure S10: MS of TMS derivative compound **8a**, Figure S11: TLC of extracts from selected *Drosera* species, Table S1: MS data of identified naphthoquinone derivatives.
