# Supplementary material for: Contrasting Dihydronaphthoquinone Patterns in Closely Related Drosera (Sundew) Species Enable Taxonomic Distinction and Identification
Source: Plants (Basel). 2021 Aug 4;10(8):1601. doi: 10.3390/plants10081601 (PMC8400376; doi:10.3390/plants10081601)
Supplement: Supplementary file 1 [file plants-10-01601-s001.zip › plants-1314927-supplementary.pdf]

Contrasting Dihydronaphthoquinone Patterns in Closely Related *Drosera* (Sundew) Species Enable Taxonomic Distinction and Identification

Jan Schlauer[1], Siegfried R. H. Hartmeyer[2], Irmgard Hartmeyer[2], Tuulikki Seppänen-Laakso[3], Heiko Rischer[3]

Affiliation

[1] University of Tuebingen, ZMBP, Auf der Morgenstelle 32, D-72076 Tuebingen, Germany

[2] Wittlinger Str. 5, D-79576 Weil am Rhein, Germany

[3] VTT Technical Research Centre of Finland Ltd., Tietotie 2, FIN-02044 Espoo, Finland

Correspondence

Jan Schlauer

University of Tuebingen, ZMBP

Auf der Morgenstelle 32

D-72076 Tübingen

Germany

jan@carnivorousplants.org

Heiko Rischer

VTT Technical Research Centre of Finland Ltd.

Tietotie 2

FIN-02044 Espoo

Finland

heiko.rischer@vtt.fi

Contents

Fig. 1S. MS of dihydroplumbagin (**5**).

Fig. 2S.MS of dihydroramentaceone (**6**).

Fig. 3S.MS of TMS derivative compound **1a**.

Fig. 4S.MS of TMS derivative compound **2a**.

Fig. 5S.MS of TMS derivative compound **3a**.

Fig. 6S.MS of TMS derivative compound **4a**.

Fig. 7S.MS of TMS derivative compound **5a**.

Fig. 8S.MS of TMS derivative compound **6a**.

Fig. 9S.MS of TMS derivative compound **7a**.

Fig. 10S.MS of TMS derivative compound **8a**.

Fig. 11S. TLC of extracts from selected *Drosera* species

Table 1S. MS Data of Identified Naphthoquinone Derivatives

Fig. 1S. MS of dihydroplumbagin (**5**).

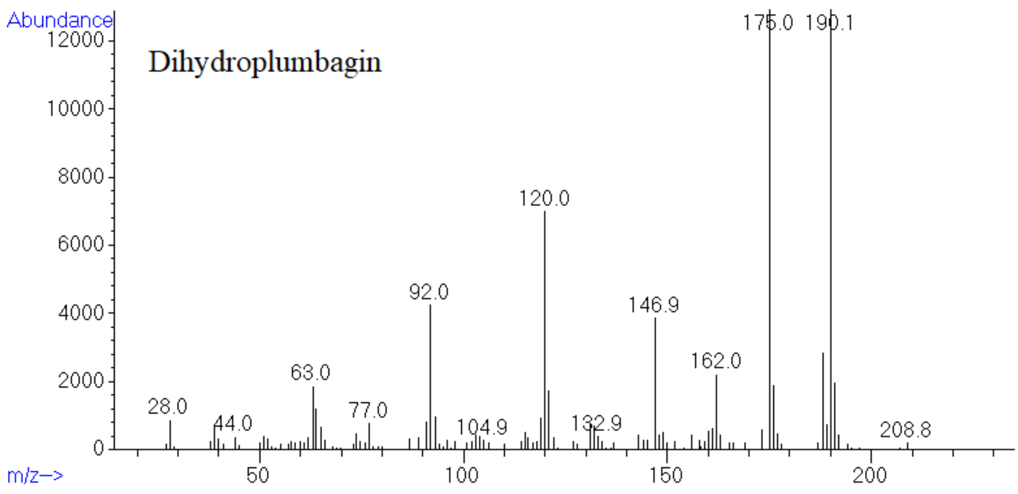

Fig. 2S. MS of dihydroramentaceone (**6**).

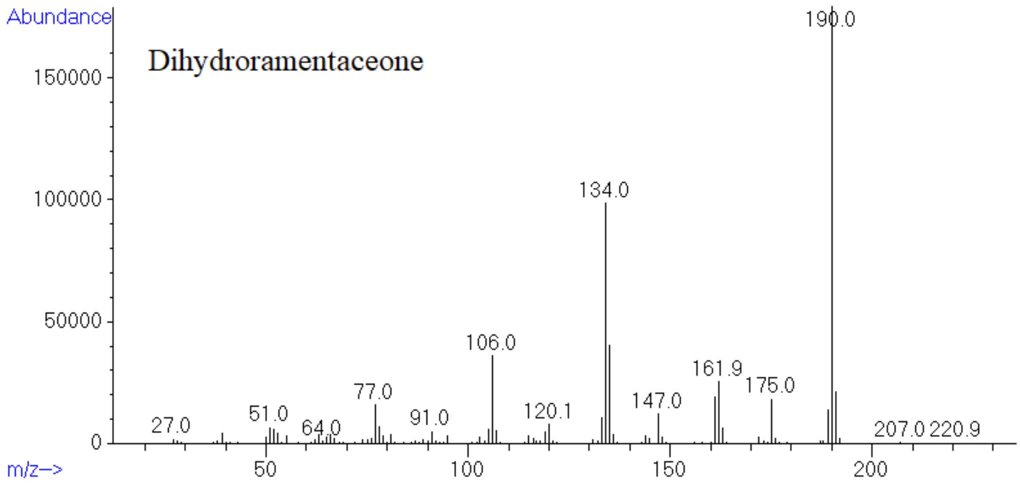

Fig. 3S. MS of TMS derivative compound **1a**.

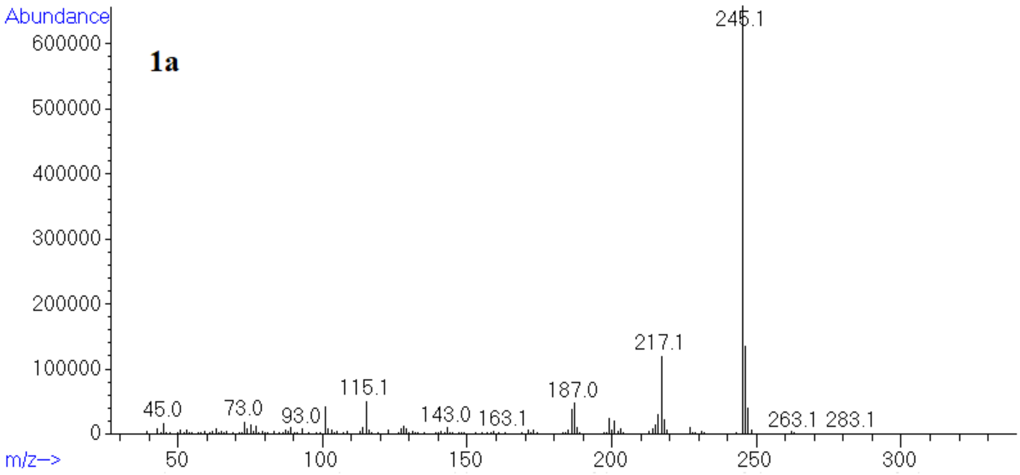

Fig. 4S. MS of TMS derivative compound **2a**.

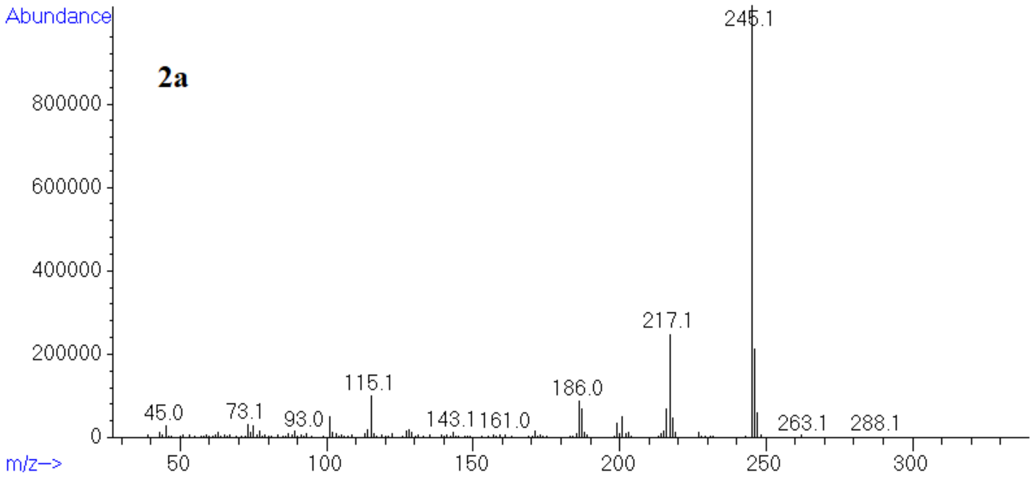

Fig. 5S. MS of TMS derivative compound **3a**.

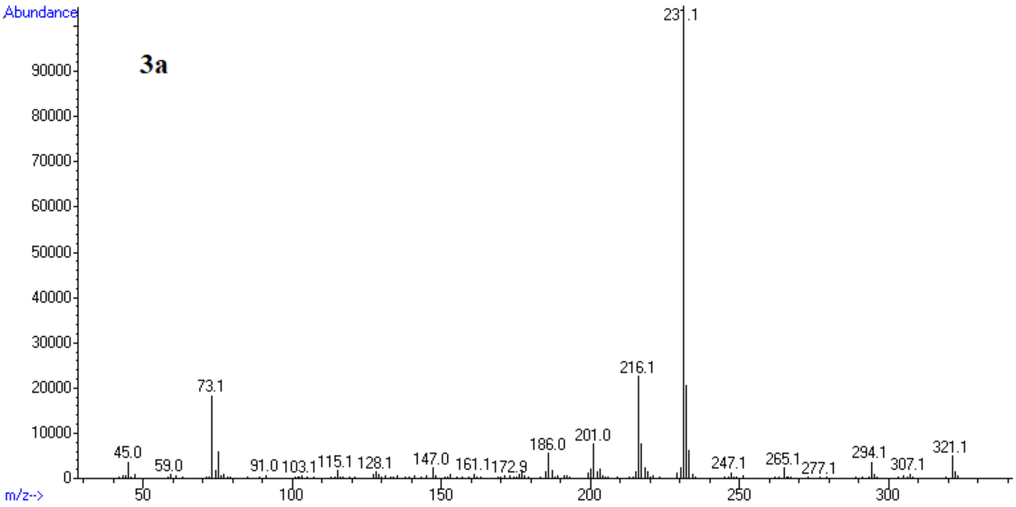

Fig. 6S. MS of TMS derivative compound **4a**.

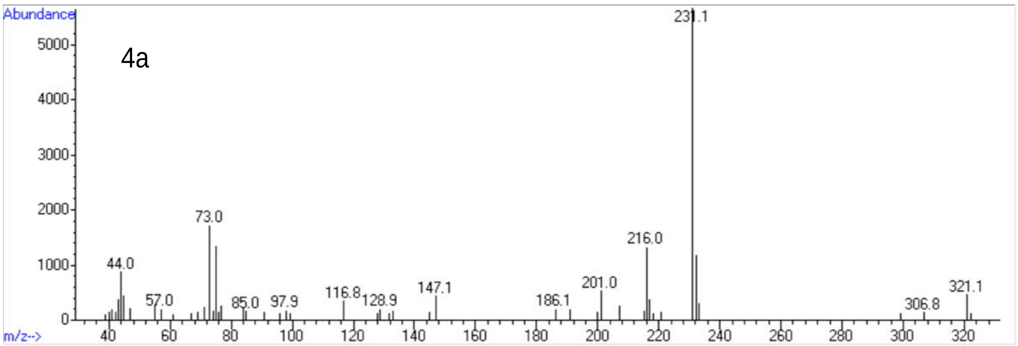

Fig. 7S. MS of TMS derivative compound **5a**.

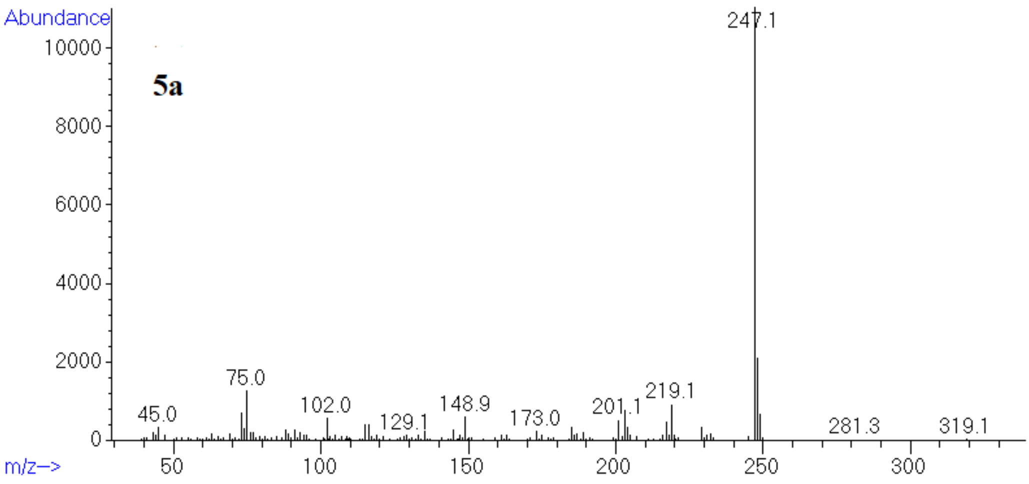

Fig. 8S. MS of TMS derivative compound **6a**.

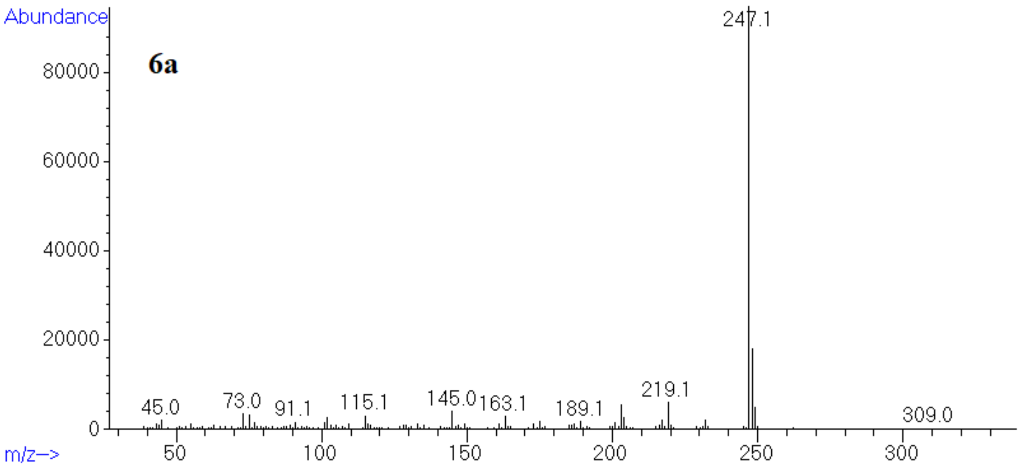

Fig. 9S. MS of TMS derivative compound **7a**.

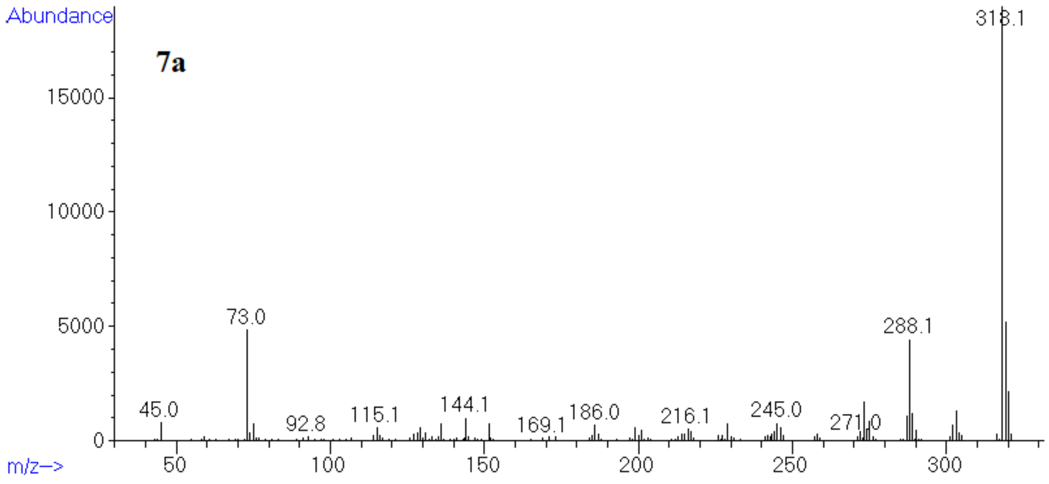

Fig. 10S. MS of TMS derivative compound **8a**.

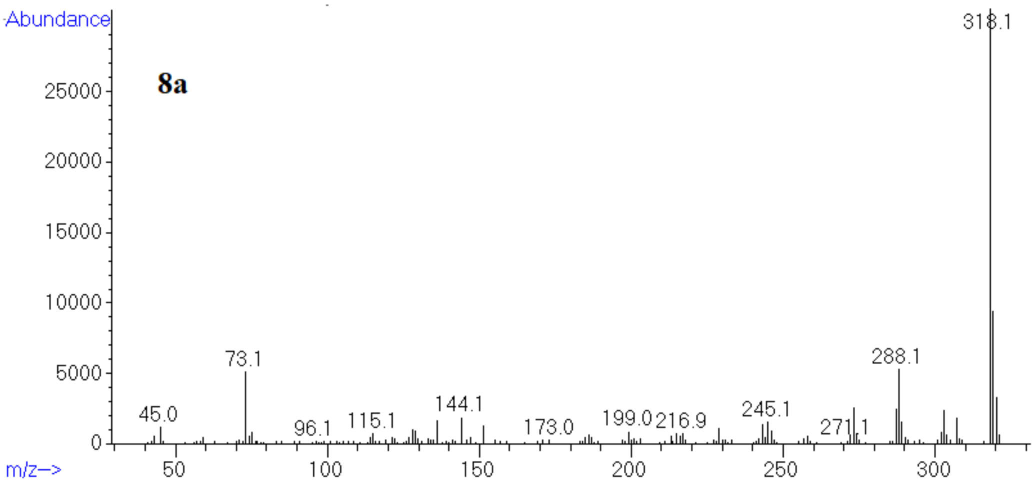

Fig. 11S. TLC of extracts from selected *Drosera* species

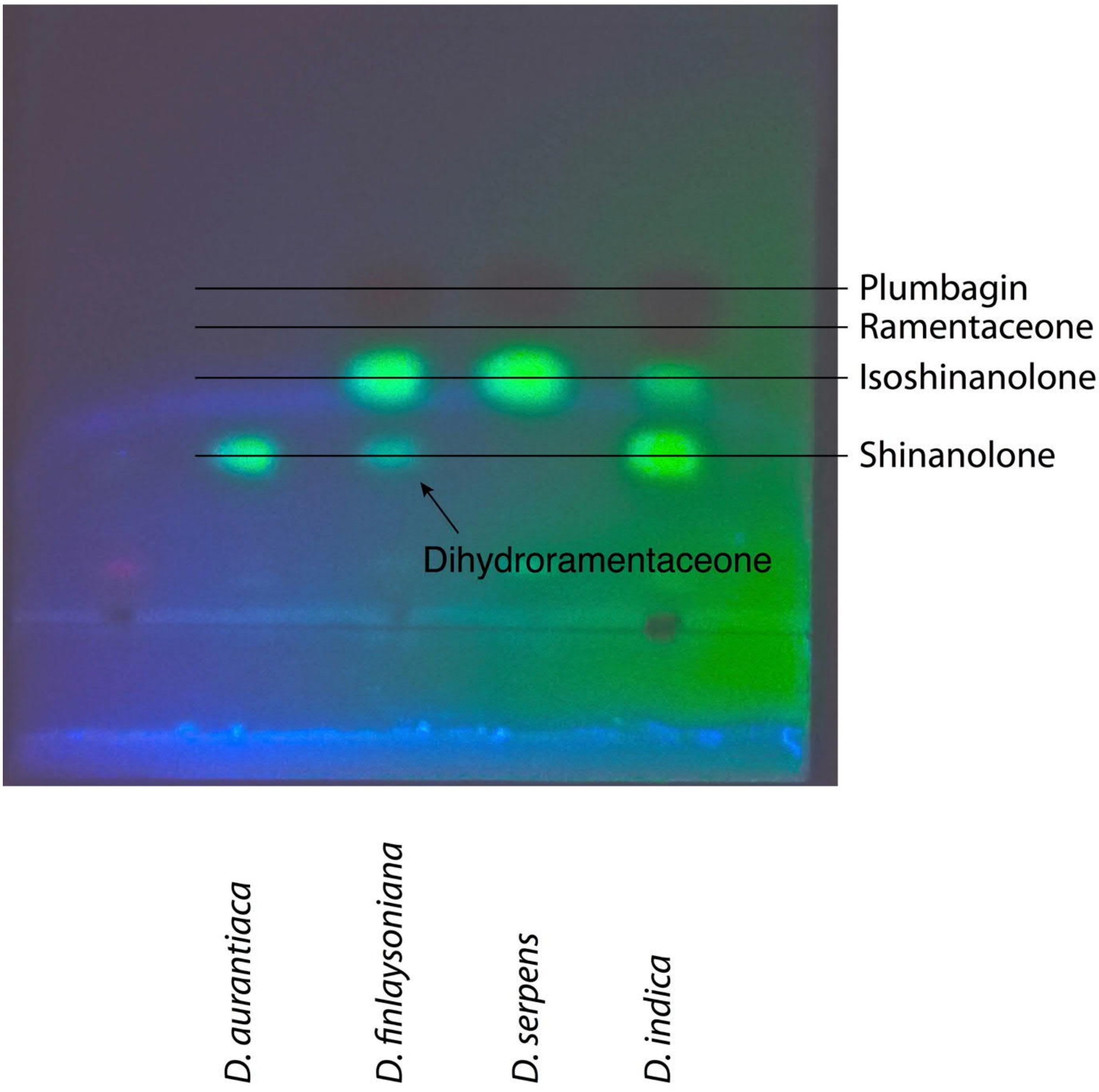

Table 1S. MS Data of Identified Naphthoquinone Derivatives

Name (**no.**)	Molecular formula	EIMS *m/z* (%) [interpretation]
Plumbagin (**1**)	$C_{11}H_8O_3$	188 $[M]^+$ (100), 173 $[M-CH_3]^+$ (30), 160 $[M-CO]^+$ (25), 132 (30), 131 $[M-C_3H_5O]^+$ (50), 121 $[M-C_4H_3O]^+$ (15), 120 $[M-C_4H_4O]^+$ (25), 103 $[M-C_4H_5O_2]^+$ (10), 92 $[M-C_5H_4O_2]^+$ (40), 77 (15), 63 (40)
Ramentaceone (**2**)	$C_{11}H_8O_3$	188 $[M]^+$ (100), 187 $[M-H]^+$ (30), 173 $[M-CH_3]^+$ (10), 160 $[M-CO]^+$ (15), 134 $[M-C_3H_2O]^+$ (20), 132 (30), 131 $[M-C_3H_5O]^+$ (30), 106 $[M-C_4H_2O_2]^+$ (20), 104 (10), 103 $[M-C_4H_5O_2]^+$ (10), 78 (15), 77 (25), 63 (15), 62 (10), 51 (25)
Isoshinanolone (**3**)	$C_{11}H_{12}O_3$	192 $[M]^+$ (70), 177 $[M-CH_3]^+$ (20), 174 $[M-H_2O]^+$ (10), 150 $[M-C_3H_6]^+$ (40), 149 $[M-C_2H_3O]^+$ (25), 131 $[M-C_2H_5O_2]^+$ (20), 122 $[M-C_3H_2O_2]^+$ (45), 121 $[M-C_4H_7O]^+$ (100), 115 (10), 93 (25), 77 (20), 65 (30), 51 (20)
Shinanolone (**4**)	$C_{11}H_{12}O_3$	192 $[M]^+$ (70), 177 $[M-CH_3]^+$ (10), 174 $[M-H_2O]^+$ (10), 164 $[M-C_2H_4]^+$ (20), 149 $[M-C_2H_3O]^+$ (15), 135 $[M-C_3H_5O]^+$ (100), 107 $[M-C_4H_5O_2]^+$ (20)
Dihydroplumbagin (**5**)	$C_{11}H_{10}O_3$	190 $[M]^+$ (85), 175 $[M-CH_3]^+$ (100), 162 $[M-CO]^+$ (20), 147 $[M-C_2H_3O]^+$ (20), 120 $[M-C_4H_6O]^+$ (60), 92 $[M-C_5H_6O_2]^+$ (40), 63 (15)
Dihydroramentaceone (**6**)	$C_{11}H_{10}O_3$	190 $[M]^+$ (100), 175 $[M-CH_3]^+$ (10), 162 $[M-CO]^+$ (15), 134 $[M-C_3H_4O]^+$ (55), 106 $[M-C_4H_4O_2]^+$ (20)
5-O-Trimethylsilyl-plumbagin (**1a**)	$C_{14}H_{16}O_3Si$	245 $[M-CH_3]^+$ (100), 217 $[M-CH_3-CO]^+$ (30), 186 (10), 115 (10)
5-O-Trimethylsilyl-ramentaceone (**2a**)	$C_{14}H_{16}O_3Si$	245 $[M-CH_3]^+$ (100), 217 $[M-CH_3-CO]^+$ (20), 187 (10), 115 (10)
4,8-Di-(O-trimethylsilyl)-isoshinanolone (**3a**)	$C_{17}H_{28}O_3Si_2$	321 $[M-CH_3]^+$ (5), 231 $[M-CH_3-C_3H_{10}OSi]^+$ (100), 216 $[M-2CH_3-C_3H_{10}OSi]^+$ (20), 201 $[M-3CH_3-C_3H_{10}OSi]^+$ (10), 186 $[M-4CH_3-C_3H_{10}OSi]^+$ (5)
4,8-Di-(O-trimethylsilyl)-shinanolone (**4a**)	$C_{17}H_{28}O_3Si_2$	321 $[M-CH_3]^+$ (5), 231 $[M-CH_3-C_3H_{10}OSi]^+$ (100), 216 $[M-2CH_3-C_3H_{10}OSi]^+$ (20), 201 $[M-3CH_3-C_3H_{10}OSi]^+$ (10), 186 $[M-4CH_3-C_3H_{10}OSi]^+$ (5)
5-O-Trimethylsilyl-dihydroplumbagin (**5a**)	$C_{14}H_{18}O_3Si$	247 $[M-CH_3]^+$ (100), 219 $[M-CH_3-CO]^+$ (10)
5-O-Trimethylsilyl-dihydroramentaceone (**6a**)	$C_{14}H_{18}O_3Si$	247 $[M-CH_3]^+$ (100), 219 $[M-CH_3-CO]^+$ (10)
1,5-Di-(O-trimethylsilyl)-2-methyl-naphtho-1,4,5-triol (**7a**)	$C_{17}H_{26}O_3Si_2$	318 $[M-CH_4]^+$ (100), 288 $[M-CH_4-2CH_3]^+$ (20), 273 $[M-CH_4-3CH_3]^+$ (10)
4,5-Di-(O-trimethylsilyl)-2-methyl-naphtho-1,4,5-triol (**7b**)	$C_{17}H_{26}O_3Si_2$	319 $[M-CH_3]^+$ (100), 245 (15), 217 (10)
2-Methyl-1,4,5-tri-(O-trimethylsilyl)-naphtho-1,4,5-triol (**7c**)	$C_{20}H_{34}O_3Si_3$	406 $[M]^+$ (100)
1,5-Di-(O-trimethylsilyl)-7-methyl-naphtho-1,4,5-triol (**8a**)	$C_{17}H_{26}O_3Si_2$	318 $[M-CH_4]^+$ (100), 288 $[M-CH_4-2CH_3]^+$ (20), 273 $[M-CH_4-3CH_3]^+$ (10)
4,5-Di-(O-trimethylsilyl)-7-methyl-naphtho-1,4,5-triol (**8b**)	$C_{17}H_{26}O_3Si_2$	319 $[M-CH_3]^+$ (100), 245 (15), 217 (10)
7-Methyl-1,4,5-tri-(O-trimethylsilyl)-naphtho-1,4,5-triol (**8c**)	$C_{20}H_{34}O_3Si_3$	406 $[M]^+$ (100)
